# An innovative approach for serial injection in marginal vein and blood collection from auricular artery in New Zealand white Rabbit

**DOI:** 10.1016/j.mex.2017.11.001

**Published:** 2017-11-11

**Authors:** Prathap Moothamadathil Baby, Sanu Susan Jacob, Rajesh Kumar, Pramod Kumar

**Affiliations:** a Department of Physiology, Melaka Manipal Medical College (Manipal campus), Manipal University, Manipal, Karnataka, India; b Department of Physiology, Kasturba Medical College, Manipal University, Manipal, Karnataka, India; c Department of Radiotherapy Oncology, Kasturba Medical College, Manipal University, Manipal, Karnataka, India; d Department of Plastic surgery, Kasturba Medical College, Manipal University, Manipal, Karnataka, India

**Keywords:** Serial injection and blood collection, Serial, Auricular artery, Marginal vein, Cannula

## Abstract

Serial injection into marginal vein and blood collection through auricular artery in New Zealand white Rabbit (Oryctolagus cuniculus) is an important procedure for various types of experimental studies. Limitations of the existing methods for serial injection and blood collection includes complex procedures, causes considerable discomfort to rabbits, whole blood samples obtained are highly prone to hemolysis and lastly detailed protocol is not available in the literature.

Approximately 10 min before commencement of the experiment, a local anesthetic cream was applied over the right and left ear lobes. The skin at the site of sample collection and injection was prepared by shaving the area on both ears and wiping it with alcohol swab. Once prepared, a 26 GA (BD Neoflon) intravenous cannula was inserted into the marginal vein of the ear and secured with an adhesive plaster. In the other ear, a 24 GA (BD Neoflon) intravenous cannula was placed in the auricular/central artery and secured with an adhesive plaster.

The novel and refined method described here has been standardized and found to be reliable. The samples obtained using this method is not susceptible to hemolysis and hence we recommend this method for serial injection and blood collection in rabbits.

• Easy to perform
• Not prone to hemolysis
• Detailed methodology described

## Graphical abstract

**Figure:**
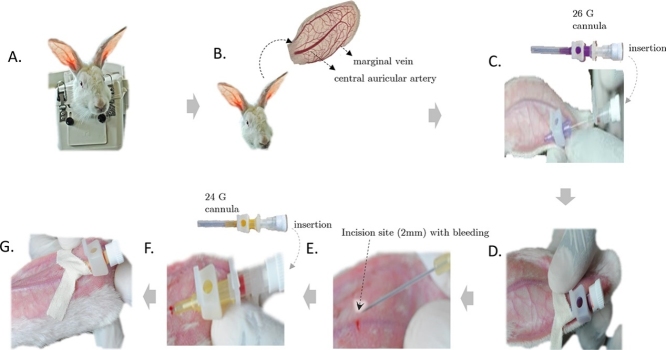

## Method details

Serial injections of anaesthesia, hormones, metabolites, drugs and repetitive blood sample collections constitute an important competency for rabbit physiological experimental studies [1], [2], [3]. Arterial and venous vessels are prominent and thus easily accessible in a rabbit ear, making it conducive for blood volume determinations. However, detailed protocols on serial injections into rabbit marginal vein and repeated large blood volume samplings from rabbit auricular artery are currently lacking [4], [5], [6], [7].

The pre-existing methodologies described in the literature as early as 1951 to 2010 are complex to perform and cause considerable discomfort to animals. Further, the blood samples obtained are highly prone to hemolysis rendering invalid results. In addition, precise methodological details and lack of accurate protocol, limits their utility for experiments [4], [5], [6], [7]. We describe here a novel technique for serial injection and blood sample collection in a rabbit model.

The animals experimental protocol were approved by the research committee of the institute (IAEC/KMC/07/2007-2008). New Zealand white rabbits (*Oryctolagus cuniculus)*, (n = 35; male n = 12, female n = 23), over 2 months of age with average 2.18 kg weight were used for the study. A 12:12 h light dark cycle with 28 °C and 30–70% relative humidity were maintained in the animal housing. Two rabbits of the same sex were hygienically housed in a standard stainless-steel cage and the collection pans were cleaned daily with water. The rabbits were fed with food and water adlibitum, except during the experiment.

The materials required for serial blood sample injection and collection includes: rabbit, rabbit restrainer, local anesthetic agent, size 11 sterile surgical blades, cotton, 95% v/v alcohol, BD Neoflon™ 24 GA IV cannula (0.7 mm × 19 mm), BD Neoflon™ 26 GA IV cannula (0.6 mm × 19 mm), surgical adhesive plaster, 5 ml syringe and blood collection tubes.

**Fig. 1:**
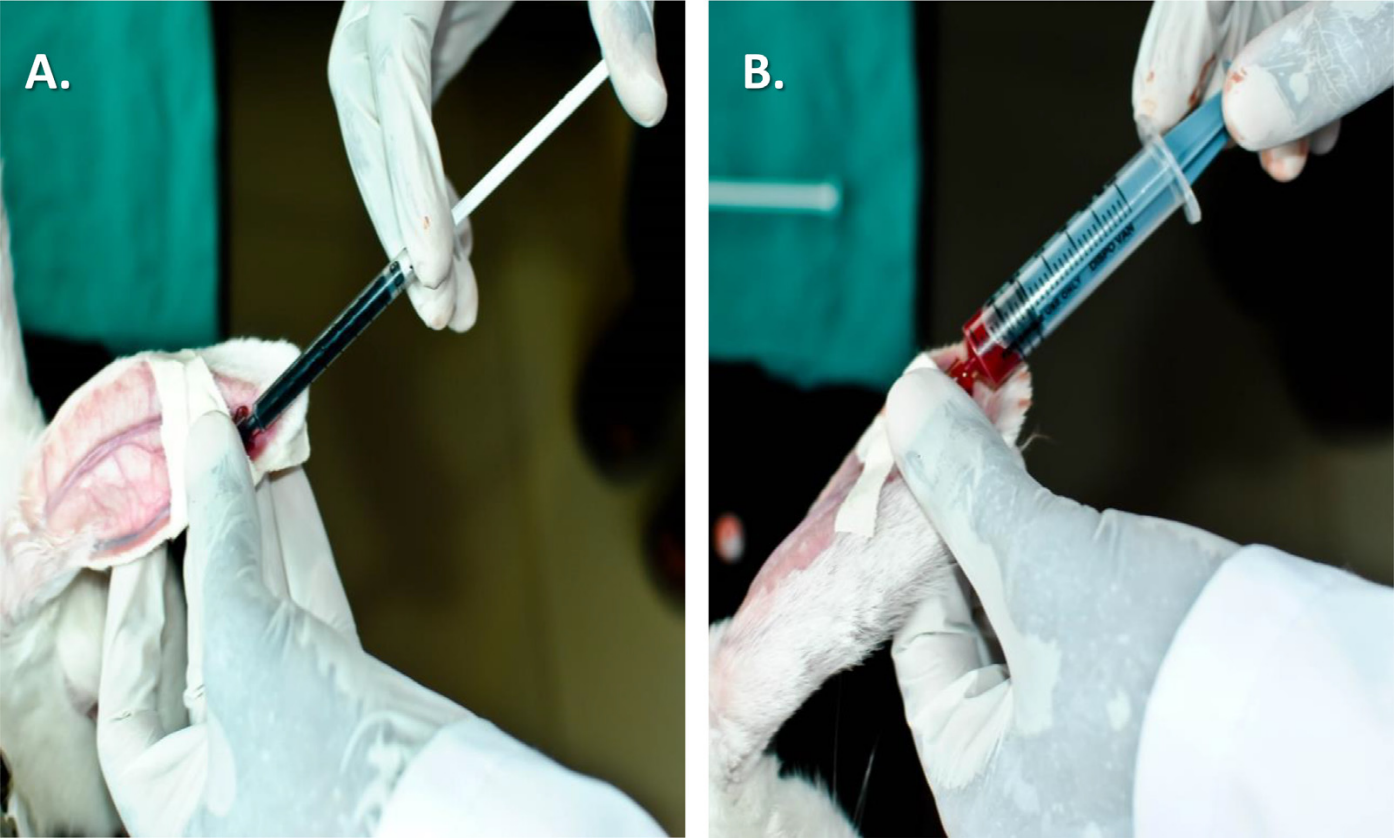
A) Injection of Evan’s blue dye. B) Collection of blood from auricular artery.

Rabbits were restrained throughout the experimental procedure. The right ear rabbit marginal vein was used for intravenous injections whereas the left ear rabbit auricular artery for repeated large blood volume samplings. A local anesthetic EMLA^®^CREAM (lidocaine 2.5% and prilocaine 2.5%) was applied and retained for 10 min over right and left rabbit dorsal ears lobes. Both the sites were prepared by shaving the area with a size 11 sterile surgical blade (Lister Surgical CO.) and gently wiping it with 95% v/v alcohol swab. Ear blood vessels are ensured visible, if not, gentle tap given to ensure engorgement. As the vessels appear prominently, a 26 GA intravenous cannula (BD Neoflon™ 26 GA IV cannula 0.6 mm × 19 mm) with the needle bevel facing up, was inserted parallel to the marginal vein of the right ear. A rush of blood into the cannula would be observable if the cannula gets correctly lodged into the marginal vein. Gentle finger pressure was applied distal to the catheter, to cease blood flow and the needle was pulled apart. The cannula was capped with Luer lock plug and secured with a surgical adhesive plaster in a loop form. This route can be used for serial injection of substances like anesthesia, hormones, metabolites and drugs for experimental studies into the rabbit vascular system (Fig. 1A).

Similarly, for obtaining large volumes of blood from the left ear auricular artery, a 2 mm wide incision was made with care using a size 11 sterile surgical blade (Lister Surgical CO.). It was made sure to avoid inadvertent damage to the artery during incision. Incision ensured easy cannulation across the superficial skin and underlying fascia over the auricular artery region. Subsequently, with the needle bevel facing up and parallel to the auricular artery a BD Neoflon™ 24 GA intravenous cannula (0.7 mm × 19 mm) was inserted through the small incision. As the cannula was positioned into the auricular artery, owing to high arterial pressure, a rush of blood ensues, which need to be attentively limited by applying finger pressure, capping cannula with Luer lock plug and secured with a surgical adhesive plaster in a loop form.

For collection of blood through the cannula, finger pressure was applied on the artery distal to the catheter, Luer lock plug of the cannula was released and cannula connected with a 5 ml syringe to obtain requisite blood volume (Fig. 1B). Slow suction pressure was essential to avoid collapse of the auricular artery and thus to avoid hemolysis of blood collected. This can be repeated for serial blood sample collection.

12 ml blood was collected over a duration of 35 min, with an initial 3.2 ml withdrawal followed by four samples of 2.2 ml each at 8 min interval.

Serial blood collection is a very important procedure in biomedical research. Minor error in obtaining blood sample leads to invalid results [7]. The cost of the above-mentioned procedure is estimated to be INR 300. Occasionally, the artery may constrict during the collection process, shutting down the flow of blood. To avoid this, gentle massage over the ear lobe can be done to enhance continuous blood flow.

Occlusion owing to blood clots, during prolonged arterial collection and venous injections, can be overcome by flushing the catheter with 0.1 ml heparin lock flush solution (Heparin Sodium, Hep-Flush-10, 10 U/ml, APP Pharmaceuticals) to ensure patency of cannula.

The blood volume of New Zealand white rabbits was estimated using this procedure, by injection of Evan’s blue dye into the marginal vein and serial blood samples were obtained from the auricular artery to estimate the concentration of the dye [8]. It has been observed that the mean blood volume values using this procedure (65.76 ml/kg) falls within the normal physiological range (45–70 ml/kg) in New Zealand white rabbits [5].

The novel method described here can be easily performed, is economical and reliable. Since the samples obtained using this method is less susceptible to hemolysis, we recommend this for serial injection and blood collection in rabbits.
